# Large-Scale Large Language Model (LLM)-Assisted Report Mining for Elbow Tendon Co-occurrence Epidemiology: Prevalence, Association, and Validation

**DOI:** 10.7759/cureus.114977

**Published:** 2026-08-22

**Authors:** Kian A Huang, Sonia V Prakash, Haris K Choudhary, Neelesh S Prakash

**Affiliations:** 1 Diagnostic Radiology, University of South Florida Morsani College of Medicine, Tampa, USA

**Keywords:** common extensor tendon, distal biceps tendon, elbow mri, large language model, prevalence

## Abstract

Introduction: Distal biceps tendon pathology and common extensor (lateral epicondylar) tendon pathology are frequent causes of chronic elbow pain and may arise from overlapping degenerative or overuse mechanisms. However, the frequency with which these two entities coexist on elbow MRI has not been well characterized in a large imaging cohort. This study aimed to determine the prevalence of and association between distal biceps pathology and common extensor pathology on elbow MRI using a large language model (LLM)-assisted radiology report extraction pipeline and to evaluate the pipeline's reliability against independent human review.

Methods: In this retrospective cross-sectional observational study, 51,169 upper-extremity MRI reports were queried from an institutional dataset from 2025 to 2026. A broad, non-pathology-filtered elbow MRI cohort was identified using procedure metadata, yielding 10,222 examinations. Reports were classified for distal biceps pathology and common extensor/lateral epicondylar pathology using a locally deployed LLM (Qwen2.5 7B Instruct via Ollama; Alibaba Cloud (Qwen Team, Alibaba Group), Hangzhou, China) with a structured, negation-aware prompt and constrained JavaScript Object Notation (JSON) output. Prevalence was estimated with Wilson confidence intervals (CIs), the association was assessed with the chi-square (χ²) test and summarized as a prevalence ratio, and effect magnitude was evaluated with Cramér's V (φ). To evaluate extraction reliability, 400 reports were randomly sampled for blinded independent human review, with results compared against LLM classifications to estimate agreement.

Results: Of 10,222 reports, 10,086 (98.7%) were successfully classified by the model. Distal biceps pathology was present in 1,301 examinations (12.7%; 95% CI: 12.1-13.4%), and common extensor pathology was present in 3,818 examinations (37.4%; 95% CI: 36.4-38.3%), with both present concurrently in 696 examinations. Distal biceps pathology was significantly associated with a higher prevalence of concomitant common extensor pathology (prevalence ratio: 1.53; χ² = 165.3, p < 0.001), though the association was modest (φ = 0.127). Human validation of 400 reports demonstrated substantial agreement for both variables (distal biceps: accuracy 84.2%, κ = 0.685; common extensor: accuracy 85.2%, κ = 0.705). Classification errors were balanced for distal biceps pathology, but the model systematically over-classified common extensor pathology (sensitivity 96.1%, specificity 78.5%, McNemar p < 0.001), indicating that the reported common extensor prevalence is likely a modest overestimate.

Conclusion: Distal biceps pathology on elbow MRI is associated with a modestly increased prevalence of concomitant common extensor pathology. However, because the extraction pipeline systematically over-classified common extensor pathology relative to human review, this association is more likely overestimated than underestimated; a shared degenerative or biomechanical mechanism therefore remains only one plausible explanation. Large-scale LLM-assisted report extraction is a feasible adjunct to manual review for musculoskeletal imaging epidemiology, though bias-adjusted, externally validated estimates are necessary before these findings are considered definitive.

## Introduction

Distal biceps tendon pathology and common extensor (lateral epicondylar) tendon pathology are among the most frequently encountered causes of chronic elbow pain in both athletic and general populations [1]. Distal biceps abnormalities span a spectrum from tendinosis and insertional degeneration to partial and full-thickness tearing, while common extensor pathology encompasses a range of chronic degenerative changes often described clinically as lateral epicondylitis or lateral epicondylopathy [2, 3]. Both entities have been attributed to repetitive loading, chronic overuse, and age-related tendon degeneration [3-5].

MRI is central to the evaluation of chronic elbow pain because it allows direct characterization of tendon morphology, intrasubstance signal abnormality, partial- or full-thickness tearing, and adjacent soft tissue change [6]. Although distal biceps and common extensor pathology may share overlapping biomechanical and degenerative pathways, the degree to which they co-occur on MRI has not been well established in a large, unselected imaging population.

Prior work examining tendon co-occurrence patterns in musculoskeletal imaging has generally relied on manual chart or report review, which limits achievable sample size and is vulnerable to inconsistent terminology extraction from free text reports. Large language models (LLMs) offer a scalable alternative for extracting clinically relevant findings from unstructured radiology text, with greater tolerance for terminology variation and surrounding context than rule-based natural language processing [7, 8]. However, because LLM output can be inconsistent or occasionally inaccurate, any epidemiologic conclusions drawn from LLM-extracted data should be corroborated against independent human review; as reported below, this corroboration step surfaced a clinically important, directional misclassification pattern for one of the two extracted variables.

## Materials and methods

Study design and data source

This retrospective cross-sectional observational study, conducted at Tampa General Hospital, Tampa, FL, USA, analyzed de-identified radiology reports from consecutive upper-extremity MRI examinations interpreted between 2025 and 2026 through the nationwide reporting database of Radiology Partners, a private radiology practice providing imaging interpretation services for hospitals and outpatient imaging centers across the United States. This proprietary internal database has no public repository, and the raw data is not publicly shareable. The study cohort, therefore, represented examinations performed at multiple institutions nationwide rather than a single center. Because distal biceps pathology and common extensor pathology were assessed simultaneously from each examination without longitudinal follow-up, the study estimated the prevalence and co-occurrence of these findings rather than incidence or future risk. The study involved no direct patient contact. All analyses were performed in Python 3 (Python Software Foundation, Wilmington, DE, USA), with report text stored in comma-separated value (CSV) format and processed using the pandas library.

Cohort selection

A broad initial MRI cohort was identified from procedure metadata using regular-expression matching for elbow- and adjacent-anatomy terminology, including “MRI elbow,” “elbow MRI,” “elbow,” “upper extremity,” “humerus,” and “forearm.” This intentionally broad, non-pathology-specific strategy was designed to maximize sensitivity for examinations that could contain the anatomic regions of interest, particularly the distal biceps tendon and common extensor/lateral epicondylar region, while accounting for variation in procedure naming conventions across contributing institutions.

The initial metadata search was followed by a second-stage review of the radiology report text to further verify that examinations were relevant to the elbow and the anatomic regions of interest. Report findings were screened for the presence of “elbow,” “bicipital tendon”, and relevant terminology variants, and “lateral epicondyle” and related terminology. This additional report-level filtering was used to distinguish true elbow-related examinations from studies captured by the intentionally broad metadata search. The broader search initially captured some non-elbow examinations, most commonly shoulder MRI examinations, which were excluded during this verification process. Only examinations meeting the criteria for an elbow-related MRI with potential visualization of the distal biceps and/or lateral epicondylar region were retained. This process yielded 10,222 elbow-related MRI examinations for downstream analysis.

Natural language processing pipeline

LLM Architecture

Report classification was performed with a locally deployed LLM (Qwen2.5 7B Instruct; Alibaba Cloud (Qwen Team, Alibaba Group), Hangzhou, China) served via Ollama, queried through a locally hosted Representational State Transfer (REST) application programming interface (API) using the Python requests library, with graphics processing unit (GPU)-accelerated local inference.

Report Preprocessing

Null reports were removed, text was converted to a standardized string format, whitespace was normalized, and indices were reset to preserve deterministic report alignment. Reports were processed in batches of three, using a ThreadPoolExecutor with three concurrent workers to balance throughput against JavaScript Object Notation (JSON) output stability.

Prompt Design

The model independently classified two binary variables per report: (1) distal biceps pathology and (2) common extensor/lateral epicondylar pathology. The prompt incorporated an extensive, pre-specified terminology list for each variable. For distal biceps pathology, this list included terms such as tendinosis, tendinopathy, tendinitis, degeneration, fraying, insertional pathology, partial tear, high-grade tear, rupture, and fiber disruption. For common extensor pathology, the list included lateral epicondylitis, lateral epicondylopathy, common extensor tendinosis, extensor origin tearing, extensor carpi radialis brevis pathology, and insertional tendinopathy. The prompt also incorporated explicit negation-handling instructions covering terms such as intact, normal, unremarkable, no tear, without abnormality, and negative for. The model was instructed to evaluate the Findings and Impression sections jointly and to avoid inferential classification when pathology status was ambiguous.

The prompt used was the following: "You are an expert musculoskeletal radiology extraction system. Your task is to classify each MRI report independently, and you must output only a valid JSON array. Output only valid JSON with no markdown, no explanations, no comments, and no text before or after the JSON. Use only double quotes and lowercase true or false. Provide one JSON object per report, and use the exact report ID provided. You must determine two variables for each report: distal_biceps_pathology and common_extensor_pathology. Distal biceps pathology should be classified as true if any of the following are present: distal biceps tendinosis, tendinopathy, tendinitis, degeneration, degenerative change, fraying, abnormality, partial tear, low-grade tear, high-grade tear, split tear, rupture, full thickness tear, attenuation, thickening, edema, strain, insertional pathology, insertional tendinopathy, insertional degeneration, insertional tearing, insertional fraying, bicipitoradial bursitis associated with distal biceps disease, reactive edema surrounding the distal biceps tendon, abnormal distal biceps tendon signal, heterogeneity, irregularity, fiber disruption, or delamination. Include chronic, postoperative, degenerative, mild, low-grade, healed, or chronic disease if currently described on MRI. Common extensor or lateral epicondylar pathology should be classified as true if any of the following are present: lateral epicondylitis, lateral epicondylopathy, common extensor tendinosis, tendinopathy, tendinitis, degeneration, degenerative change, fraying, abnormality, partial tear, low-grade tear, high-grade tear, split tear, rupture, full thickness tear, extensor tendon tear, extensor tendon tendinosis, tendinopathy, degeneration, fraying, abnormality, fiber disruption, heterogeneity, irregularity, extensor carpi radialis brevis pathology, ECRB tendinosis, tear, degeneration, tennis elbow, lateral elbow tendinopathy, lateral elbow common extensor pathology, lateral condylar tendinopathy, lateral extensor origin pathology, extensor origin degeneration, tearing, tendinopathy, or tendinosis. Include chronic, postoperative, degenerative, mild, low-grade, healed, or chronic disease if currently described on MRI. Classify each variable as false if the report states no tear, no tendinosis, no tendinopathy, intact, normal, unremarkable, without abnormality, no evidence of, negative for, without pathology, no distal biceps abnormality, no common extensor abnormality, distal biceps tendon intact, or common extensor tendon intact, unless pathology is explicitly stated elsewhere. Use both the findings and impression sections of the report. Do not infer absent findings, do not hallucinate pathology, and both variables may be true simultaneously. If you are uncertain, classify the finding as false. Current MRI findings override historical wording; historical symptoms alone do not count, and prior surgery counts only if a current abnormality exists."

Output Parsing

The model was constrained to return a JSON array with a fixed schema per report (report identifier and two boolean fields). Custom parsing logic extracted bracketed JSON content from each response; malformed responses triggered automated retry. Reports that failed to parse after retry were defaulted to a negative classification for both variables and flagged as unclassified.

Reliability validation

The 400 sampled reports were independently reviewed by a pre-oriented medical research staff reviewer who used a comprehensive, predefined list of pathology terminology and classification criteria. The review process was supervised by a radiologist with fellowship training in musculoskeletal imaging, who provided clinical oversight and served as the adjudicator for classification questions or uncertainties. The human reviewer was blinded to the corresponding LLM classification during independent review. Agreement between LLM and human classification was quantified separately for each variable using overall percent agreement and Cohen's kappa (κ) coefficient, interpreted according to the Landis and Koch scale, on which values of 0.61-0.80 indicate substantial agreement and 0.81-1.00 indicate almost perfect agreement [9].

Sensitivity, specificity, positive predictive value (PPV), and negative predictive value (NPV) were calculated using the human reviewer's classification as the reference standard. McNemar's test was used to determine whether discordant cases reflected a systematic directional bias (e.g., preferential over- or under-calling by the LLM) rather than symmetric, non-directional error.

Statistical analysis

Statistical analyses were performed using SciPy (SciPy community/NumFOCUS, Austin, TX, USA) and statsmodels in Python. Prevalence estimates were calculated using all 10,222 examinations in the elbow MRI cohort as the denominator. The 136 reports (1.3%) that could not be classified by the LLM pipeline after retry were retained in the analytic cohort and defaulted to negative for both distal biceps and common extensor pathology. These examinations were therefore included in the denominators for prevalence estimates and in subsequent co-occurrence and association analyses. This prespecified handling of unclassified reports may result in modest underestimation of prevalence if pathology was present in these examinations.

Prevalence estimates are reported with 95% Wilson score confidence intervals (CIs). The association between distal biceps pathology and common extensor pathology was summarized using the prevalence ratio with corresponding 95% CIs, as the prevalence ratio provides a directly interpretable measure of association for cross-sectional prevalence data. Statistical significance of the association was assessed using the Pearson chi-square (χ²) test. Effect size was quantified using the phi (φ) coefficient. Statistical tests were two-sided, and statistical significance was defined as p < 0.05.

Sensitivity, specificity, PPV, and NPV are reported with 95% Wilson CIs. Statistical significance of directional (asymmetric) disagreement between LLM and human classification was assessed with McNemar's test, with exact binomial computation used when the number of discordant pairs was small.

## Results

Cohort characteristics

From an initial dataset of 51,169 musculoskeletal radiology reports, 10,222 elbow-related MRI examinations were identified. The LLM pipeline successfully classified 10,086 reports (98.7%); 136 reports (1.3%) could not be classified after the predefined retry procedure and were defaulted to negative for both variables (Table 1). The default-negative approach was used as a conservative failure-handling strategy to preserve the complete predefined cohort denominator rather than selectively removing reports for which the model failed to return a valid classification. Importantly, these 136 reports were not considered confirmed negative examinations; rather, negative labels were assigned solely because the classification process did not produce a valid output after retry. This approach prevented failed cases from being excluded from prevalence calculations but could result in misclassification and modest underestimation of the prevalence of either pathology if positive findings were present in the unparsed reports.

**Table 1:** Radiology report summary

Variable	Value
Initial radiology reports	51,169
Elbow MRI cohort	10,222
Successfully classified reports	10,086
Unclassified reports (defaulted negative)	136
Classification success rate	98.7%

Prevalence of tendon pathology

Distal biceps pathology was identified in 1,301 of 10,222 examinations (12.7%; 95% CI: 12.1%-13.4%). Common extensor/lateral epicondylar pathology was identified in 3,818 of 10,222 examinations (37.4%; 95% CI: 36.4%-38.3%), consistent with its recognized status as one of the most common chronic elbow tendon disorders on MRI. All 10,222 examinations were retained in the denominator for prevalence estimation, including the 136 reports (1.3%) that could not be classified by the LLM pipeline and were defaulted to negative for both pathology variables.

Co-occurrence analysis

The distribution of co-occurring pathology is summarized in Table 2. Both pathologies were present together in 696 examinations. The co-occurrence analysis included all 10,222 examinations, including the 136 reports that could not be classified and were defaulted to negative for both variables. Patients with distal biceps pathology were significantly more likely to have concomitant common extensor pathology than those without distal biceps pathology (prevalence ratio, 1.53; 95% CI: 1.44-1.62). This association was statistically significant on Pearson’s chi-square test (χ² = 165.3, p < 0.001). Effect size analysis demonstrated a statistically significant but small association (φ = 0.127), indicating that while the association is unlikely to be due to chance given the sample size, its magnitude is modest.

**Table 2:** Contingency table of distal biceps and common extensor pathology

Distal biceps status	Common extensor negative	Common extensor positive
Distal biceps negative	5,799	3,122
Distal biceps positive	605	696

Reliability validation

Of the 400 randomly sampled reports, the underlying confusion matrices comparing LLM classifications with the human reviewer are shown in Table 3, with corresponding diagnostic performance metrics summarized in Table 4. For distal biceps pathology, the LLM correctly identified 165 true-positive and 172 true-negative cases, with 35 false positives and 28 false negatives (Table 3). Overall agreement was observed in 337 of 400 cases (84.2% accuracy; κ = 0.685), with sensitivity of 85.5%, specificity of 83.1%, PPV of 82.5%, and NPV of 86.0% (Table 4). McNemar's test was not significant (p = 0.45), indicating no systematic directional bias. For common extensor pathology, the LLM correctly identified 147 true-positive and 194 true-negative cases, with 53 false positives and six false negatives (Table 3). Overall agreement was 85.2% (κ = 0.705), with sensitivity of 96.1%, specificity of 78.5%, PPV of 73.5%, and NPV of 97.0% (Table 4). McNemar's test was significant (p < 0.001), indicating that the model systematically overclassified common extensor pathology relative to the human reviewer. Both variables were classified correctly in 295 of 400 cases (73.8%). Review of discordant cases with reviewer annotations identified a recurrent, interpretable source of error: several reports described findings in "the biceps" without specifying the distal portion, which the LLM and human reviewer at times interpreted differently.

**Table 3:** Confusion matrices comparing large language model classifications with the human reviewer in the validation cohort Confusion matrices comparing large-language-model-generated classifications with the reference human reviewer for the validation cohort (n = 400). Values represent the numbers of true-positive, false-positive, true-negative, and false-negative classifications and form the basis for the diagnostic performance metrics reported in Table 4.

Variable	True Positive	False Positive	True Negative	False Negative
Distal biceps pathology	165	35	172	28
Common extensor pathology	147	53	194	6

**Table 4:** Diagnostic performance and inter-rater agreement between large language model and human-reviewer classification CI = confidence interval; PPV = positive predictive value; NPV = negative predictive value (n = 400 validation cases).

Variable	Accuracy (95% CI)	Sensitivity (95% CI)	Specificity (95% CI)	PPV (95% CI)	NPV (95% CI)	Cohen’s κ
Distal biceps pathology	84.2% (80.4–87.5)	85.5% (79.8–89.8)	83.1% (77.4–87.6)	82.5% (76.6–87.1)	86.0% (80.5–90.1)	0.685
Common extensor pathology	85.2% (81.4–88.4)	96.1% (91.7–98.2)	78.5% (73.0–83.2)	73.5% (67.0–79.1)	97.0% (93.6–98.6)	0.705

## Discussion

In this large retrospective analysis of elbow MRI reports, distal biceps pathology was associated with a significantly increased likelihood of concomitant common extensor/lateral epicondylar pathology, with patients demonstrating distal biceps abnormalities showing an approximately 53% increased prevalence of common extensor pathology relative to patients without distal biceps abnormalities. As detailed below, this estimate is best treated as an upper bound, given evidence that the extraction pipeline over-classified common extensor pathology relative to human review.

Although the phi coefficient indicates that the magnitude of this association is modest, the finding is compatible with the possibility that the distal biceps and common extensor tendon complexes share overlapping degenerative or biomechanical loading patterns during elbow flexion-extension and forearm rotation [10-13]. The substantially higher prevalence of common extensor pathology relative to distal biceps pathology observed here is consistent with its established status as one of the most common chronic elbow tendon disorders; the comparatively broad, inclusive definition of distal biceps pathology used in this study, spanning degenerative change through frank tearing, likely also contributes to the difference in prevalence between the two entities [3-5, 14].

A key strength of this study is the use of a broad, non-prefiltered elbow MRI cohort, which reduces enrichment bias and improves generalizability relative to cohorts selected on the basis of a specific clinical indication [15]. The study also demonstrates the feasibility of applying a locally deployed LLM to extract clinically meaningful findings from more than 10,000 unstructured radiology reports, a scale that would be prohibitively labor-intensive with manual review, while incorporating extensive musculoskeletal terminology and explicit negation handling. This finding is consistent with a growing body of literature showing that LLM-based pipelines can approach or match rule-based and supervised NLP approaches for structured extraction from free-text radiology reports across a range of clinical domains, while requiring substantially less task-specific engineering [16, 17]. At the same time, prior evaluations of LLM-based report classification have shown that performance can be sensitive to prompt design and data variability, underscoring the importance of the blinded human reviewer validation performed here as a check on extraction reliability before the epidemiologic findings are treated as definitive [17-20].

The validation analysis supports the overall reliability of the LLM extraction pipeline, with substantial agreement (κ = 0.685-0.705) for both variables and no evidence of directional bias for distal biceps pathology. However, the pipeline systematically over-classified common extensor pathology relative to human review, which suggests the cohort-wide prevalence of common extensor pathology reported above (37.4%) is more likely an overestimate than an underestimate, while the distal biceps prevalence estimate (12.7%) is not subject to the same directional concern given its symmetric error pattern. Because the association between the two variables was defined using the same extraction pipeline, this asymmetric misclassification would be expected to bias the reported prevalence ratio, though the direction and magnitude of that bias cannot be determined without re-running the association analysis on the human-adjudicated subset. Review of individual discordant cases also identified a concrete, fixable source of error: ambiguous references to "the biceps" without the qualifier "distal," which future prompt refinement could specifically target. A straightforward next step, which we did not attempt here given the modest size of the validation sample, would be to apply the observed sensitivity and specificity to derive bias-corrected prevalence and prevalence-ratio estimates; doing so in a larger validation sample would strengthen confidence in the epidemiologic estimates reported above.

From an epidemiologic standpoint, the frequency of co-occurrence identified here illustrates the type of large-sample association that report-mining pipelines are well positioned to detect at a scale that would be impractical to achieve through manual chart review alone and suggests that distal biceps and common extensor pathology are worth evaluating jointly, rather than in isolation, when characterizing elbow tendon disease burden [16, 20-22]. Any translation of this finding into a specific recommendation about imaging thresholds or reporting practice should, however, follow rather than precede confirmation that the underlying prevalence and association estimates are not materially altered by the classification bias identified in the validation analysis; extending human-reviewer validation to a larger or externally sourced sample, and recomputing the prevalence ratio on the adjudicated subset, would be a more direct test of the robustness of this association than the unadjusted estimate reported here. Because MRI concurrently visualizes both structures within a single examination, more systematic documentation of both distal biceps and common extensor tendon status in the report, regardless of the primary clinical indication, would improve the completeness of future report-mining datasets and could support more precise, less bias-prone co-occurrence estimates in subsequent studies.

This study has several limitations. Classification was based on radiology report text rather than direct image review and therefore reflects the interpreting radiologist's documentation rather than an independent imaging reference standard. Despite extensive prompt engineering and negation handling, LLM-based extraction may still introduce classification error or fail to capture atypical terminology; the magnitude of this error is addressed by the human-review validation analysis described above. The validation reference standard was based on review by pre-oriented medical research staff using predefined pathology criteria, with supervision and adjudication by a musculoskeletal fellowship-trained radiologist. However, the validation process did not include independent dual review by multiple fellowship-trained radiologists followed by formal consensus adjudication. Accordingly, disagreement between the LLM and human classification cannot necessarily be interpreted as definitive LLM error, and the reported accuracy and κ values should be interpreted as agreement with the human review process rather than an unimpeachable ground truth. The reported prevalence ratio likewise inherits any asymmetric misclassification present in the human reference classification, particularly for common extensor pathology. The 136 reports (1.3%) that could not be parsed were defaulted to a negative classification, which could modestly underestimate true prevalence for both variables. This study reports co-occurrence and association, not causation, and a shared biomechanical or degenerative mechanism remains a plausible but unproven explanation for the observed association. Finally, data were derived from a nationwide radiology reporting database representing multiple institutions but a single radiology practice, which may limit generalizability to other populations, health systems, or practice settings.

## Conclusions

In this large, retrospective, non-pathology-prefiltered cohort of elbow MRI examinations, distal biceps pathology was associated with a significantly higher prevalence of concomitant common extensor/lateral epicondylar pathology in the LLM-derived dataset. However, this association should be interpreted cautiously given the cross-sectional study design and the demonstrated asymmetric misclassification of common extensor pathology. A blinded human-reviewer validation of 400 reports demonstrated substantial, though imperfect, agreement with the LLM extraction pipeline for both variables, supporting LLM-assisted report extraction as a feasible adjunct to, rather than a replacement for, manual review in musculoskeletal imaging epidemiology. Because common extensor pathology was significantly over-classified relative to human review, both its estimated prevalence and the observed prevalence ratio may be biased, and the reported association should therefore be considered preliminary pending further validation and bias-corrected analysis. The findings do not establish causality or demonstrate a shared biomechanical or degenerative mechanism between the two pathologies. Future work should focus on refining prompt design to address identifiable sources of classification error, including ambiguous references to bicipital abnormalities without a stated distal qualifier; evaluating prevalence and association estimates in larger, independently adjudicated validation samples; and externally validating the approach across multiple institutions and practice settings.

## References

[1] Kane SF, Lynch JH, Taylor JC (2014). Evaluation of elbow pain in adults. Am Fam Physician.

[2] Blaeser AM, Markus DH, Hurley ET, Gonzalez-Lomas G, Strauss EJ, Jazrawi LM (2021). Current controversies and decision-making in the management of biceps pathologies. JBJS Rev.

[3] Wolf JM (2023). Lateral epicondylitis. N Engl J Med.

[4] Wallis JA, Bourne AM, Jessup RL, Johnston RV, Frydman A, Cyril S, Buchbinder R (2024). Manual therapy and exercise for lateral elbow pain. Cochrane Database Syst Rev.

[5] Appiakannan HS, Kachooei AR, Ilyas AM (2023). Biceps tenodesis for distal biceps tendon repair. J Med Insight.

[6] Dewan AK, Chhabra AB, Khanna AJ, Anderson MW, Brunton LM (2013). MRI of the elbow: techniques and spectrum of disease: AAOS exhibit selection. J Bone Joint Surg Am.

[7] Cai T, Giannopoulos AA, Yu S (2016). Natural language processing technologies in radiology research and clinical applications. Radiographics.

[8] López-Úbeda P, Martín-Noguerol T, Juluru K, Luna A (2022). Natural language processing in radiology: update on clinical applications. J Am Coll Radiol.

[9] Landis JR, Koch GG (1977). The measurement of observer agreement for categorical data. Biometrics.

[10] Millar NL, Silbernagel KG, Thorborg K (2021). Tendinopathy. Nat Rev Dis Primers.

[11] Fredberg U, Stengaard-Pedersen K (2008). Chronic tendinopathy tissue pathology, pain mechanisms, and etiology with a special focus on inflammation. Scand J Med Sci Sports.

[12] Seiler JG 3rd, Parker LM, Chamberland PD, Sherbourne GM, Carpenter WA (1995). The distal biceps tendon. Two potential mechanisms involved in its rupture: arterial supply and mechanical impingement. J Shoulder Elbow Surg.

[13] Pérez-Abad M, Noriego Muñoz D, Ferreres Claramunt Á, Del Valle Jou M, Rodríguez-Baeza A (2022). The loads developed by epicondylar and epitrochlear muscles across the elbow joint. A dynamic simulated model. J Biomech.

[14] Bhatia DN, Malviya P (2024). All-endoscopic approach for distal biceps tendon pathology: analysis of long-term outcomes in partial and complete ruptures. J Shoulder Elbow Surg.

[15] Sica GT (2006). Bias in research studies. Radiology.

[16] Nowak S, Wulff B, Layer YC (2025). Privacy-ensuring open-weights large language models are competitive with closed-weights GPT-4o in extracting chest radiography findings from free-text reports. Radiology.

[17] Dorfner FJ, Jürgensen L, Donle L (2024). Comparing commercial and open-source large language models for labeling chest radiograph reports. Radiology.

[18] Talati IA, Chaves JM, Das A, Banerjee I, Rubin DL (2025). Out-of-the-box large language models for detecting and classifying critical findings in radiology reports using various prompt strategies. AJR Am J Roentgenol.

[19] Kim TT, Makutonin M, Sirous R, Javan R (2025). Optimizing large language models in radiology and mitigating pitfalls: prompt engineering and fine-tuning. Radiographics.

[20] Lee RC, Hadidchi R, Coard MC (2026). Use of large language models on radiology reports: a scoping review. J Am Coll Radiol.

[21] Thomas JM, Chang EY, Ha AS (2022). ACR Appropriateness Criteria® chronic elbow pain. J Am Coll Radiol.

[22] Rineer CA, Ruch DS (2009). Elbow tendinopathy and tendon ruptures: epicondylitis, biceps and triceps ruptures. J Hand Surg Am.

